# Unmasking Viral Causes of Hospitalized Respiratory Infection: Five Years of Respiratory Virus Surveillance in Vietnam by Multiplex Real-Time PCR Assay

**DOI:** 10.3390/v18020153

**Published:** 2026-01-23

**Authors:** Huong T. Pham, Van H. Pham, Duy K. Tran, Nhu H. T. Tran, Thao H. T. Nguyen, Anh H. Pham, Quang D. Ha, Ngoc V. Tran, Nhung V. Nguyen, Thanh V. Nguyen, Dung N. T. Nguyen, Chien D. Vo, Camelia Quek, Son T. Pham

**Affiliations:** 1 Vietnam Research and Development Institute of Clinical Microbiology, Ho Chi Minh City 70000, Vietnam; phamthienhuong@gmail.com (H.T.P.); hongnhutran9595@gmail.com (N.H.T.T.); honganhp2499@gmail.com (A.H.P.);; 2 Ho Chi Minh City Respiratory Society, Ho Chi Minh City 70000, Vietnam; 3 Faculty of Medicine, VNU University of Medicine and Pharmacy, Hanoi 10000, Vietnam; vietnhung@yahoo.com; 4 Vietnam Lung Association, Ha Noi 10000, Vietnam; 5 Ho Chi Minh City Medical Association, Ho Chi Minh City 70000, Vietnam; 6 Nguyen Tri Phuong Hospital, Ho Chi Minh City 70000, Vietnam; 7 The University of Sydney, Camperdown 2050, Australia; 8 New South Wales Health, Westmead 2145, Australia; 9 The Royal Australian College of General Practitioners, North Sydney 2060, Australia

**Keywords:** respiratory tract infection (RTI), acute lower respiratory infection (LRTI), multiplex real-time PCR (MPL real-time PCR), respiratory viruses

## Abstract

Aim of the study: To investigate the detection rate of respiratory viruses identified by multiplex real-time PCR (MPL real-time PCR) in respiratory specimens collected from hospitalized patients with acute lower respiratory tract infections (LRTI) over a five-year period (2020–2024), and to emphasize the importance of MPL real-time PCR testing in identifying respiratory viruses responsible for severe lower respiratory tract infections requiring hospitalization. Subjects and Methods: This cross-sectional retrospective study analyzed 15,936 respiratory specimens collected from hospitalized patients between 2020 and 2024. Seventeen respiratory viruses were detected using MPL real-time PCR. Statistical comparisons were performed using the chi-square test. Results and Discussion: The overall respiratory virus detection rate was 31.88% and was significantly higher in children than in adults (52.98% vs. 18.10%). The most frequently detected viruses were rhinovirus, influenza A, respiratory syncytial virus, and parainfluenza virus type 3, while influenza A and SARS-CoV-2 predominated in adults. During the peak of the COVID-19 pandemic in 2021, SARS-CoV-2 accounted for 78.92% of detected viruses, accompanied by marked suppression of other respiratory pathogens. Measles virus re-emerged in 2024, predominantly affecting children (17.65%). Most Respiratory virus-positive cases (82.8%) involved single-agent infections. Conclusions: Hospitalized acute LRTI cases often lack distinctive clinical signs to identify viral pathogens. MPL real-time PCR provides simultaneous multi-virus detection, enabling accurate etiological diagnosis and strengthening hospital-based viral surveillance, particularly in resource-limited settings.

## 1. Introduction

Respiratory viruses have been recognized as major causative agents of lower respiratory tract infection (LRTI) [1,2,3,4,5,6]. However, conventional clinical microbiology methods often fail to detect them. This is primarily because most clinical laboratories lack the capacity for viral culture. Furthermore, immunological assays are limited, as not all respiratory viruses can be identified through antigen or antibody detection. Therefore, detecting specific nucleic acid sequences of viral pathogens in lower respiratory tract specimens provides an optimal method for accurate diagnosis. Among the available molecular methods, polymerase chain reaction (PCR) offers the highest sensitivity and specificity, comparable to that of viral culture, by amplifying specific nucleic acid sequences prior to detection [7,8]. The technique has since evolved into real-time PCR, which allows simultaneous amplification and detection of target nucleic acids. This development has made the assay more practical for use in clinical microbiology laboratories as it reduces the amplicon contamination risks inherent to conventional PCR and facilitates rapid diagnosis [8].

Hospitalized cases of acute respiratory infection frequently present with nonspecific symptoms, making clinical identification of the causative pathogen difficult, especially in viral etiologies. [9,10]. Therefore, simultaneous detection of multiple respiratory viruses is essential to ensure that no respiratory pathogens are overlooked. Prior to 2020, studies in Vietnam primarily utilized in-house multiplex PCR or commercial real-time PCR assays (e.g., Seeplex™ RV Detection, Seegene) for respiratory virus surveillance [11,12,13,14,15,16]. However, in-house multiplex PCR protocols are technically challenging to implement, while commercial assays like Seegene’s are expensive and limited to a 15-virus panel. To address the clinical need for comprehensive viral detection, the Vietnam Research and Development Institute of Clinical Microbiology (VCM) developed an in-house MPL real-time PCR assay capable of detecting 18 respiratory viruses simultaneously. The target panel includes influenza A (InfA), influenza B (InfB), influenza C (InfC), parainfluenza viruses 1–3 (Para1–3), respiratory syncytial virus (RSV), human adenovirus (ADV), Measles virus (MeaVR), rhinovirus (Rhino), human metapneumovirus (hMPV), bocavirus (Boca), human coronavirus HKU1 (hCoV), SARS-CoV-2 (CoV2), SARS-CoV (SARS), MERS-CoV (MERS), varicella-zoster virus (VZV), and rubella virus (RUBV). This assay and its analytical workflow have been validated in several studies and are currently used for routine diagnostics at NK-BIOTEK Laboratory, an accredited facility fully equipped for molecular infectious disease testing [17,18,19,20].

## 2. Aims of the Study

This study aimed to determine the prevalence of respiratory viruses in hospitalized patients with acute lower respiratory tract infections (LRTI) in Vietnam from 2020 to 2024 by analyzing multiplex real-time PCR (MPL real-time PCR) results. It also sought to demonstrate the importance and feasibility of implementing the MPL real-time PCR in routine clinical microbiology laboratories.

## 3. Materials and Methods

### 3.1. Study Method

This retrospective study analyzed the MPL real-time PCR results for the detection of respiratory viruses in respiratory specimens collected from patients hospitalized with acute LRTI in Vietnam between 2020 and 2024. Data were retrieved from archived Microsoft Excel databases (version 2512) at the NK-BIOTEK Laboratory (Ho Chi Minh city, Vietnam), where comprehensive respiratory viral testing is routinely performed upon clinicians’ requests. For patients with multiple test records, only the first MPL real-time PCR result was included in the analysis; subsequent results were excluded.

### 3.2. The Samples Collection

Respiratory specimens were obtained from patients hospitalized with acute LRTI. These included sputum, bronchoalveolar lavage fluid, nasotracheal swabs, or nasotracheal aspirates. Specimens were collected immediately after clinical diagnosis of acute LRTI and transported to the laboratory for testing under cold-chain conditions (2–8 °C). For hospitals located distant from the testing laboratory, specimens were delivered within 24 h of collection.

### 3.3. Sample Preparation and MLP Real-Time PCR Testing

Sample preparation and nucleic acid extraction were performed according to the manufacturer’s instructions (Nam Khoa Company, Ho Chi Minh city, Vietnam). MPL real-time PCR was conducted by adding extracted nucleic acid to an MPL real-time PCR master mix prepared by the Vietnam Research and Development Institute of Clinical Microbiology (VCM), containing specific primers and TaqMan probes targeting four nucleic acid sequences (Table 1). Thermal cycling conditions consisted of reverse transcription at 45 °C for 10 min (RNA targets), initial denaturation and enzyme activation at 95 °C for 15 min, followed by 40 cycles of denaturation at 95 °C for 15 s and annealing at 60 °C for 1 min. Fluorescence signals were recorded during the annealing step in the FAM, HEX, Texas Red, and Cy5 channels. A target was considered positive when the Ct value was <38.5, in accordance with the manufacturer’s recommendation (Supplementary Methods).

### 3.4. Data Collection and Analysis

The data analyzed included the patient’s age (adult or pediatric), geographical location of sample collection (if possible), date of specimen collection, and MPL real-time PCR results indicating the presence or absence of respiratory viruses and, if detected, the identified viral species. For patients with multiple specimens, only the first MPL real-time PCR result was included in the analysis.

The analyses included: (i) The number of samples collected from adult and pediatric patients by year; (ii) overall respiratory viral detection rates, and, among samples positive for viral respiratory infections, the distribution of detected viruses according to age group (adult and pediatric) and, where possible, across more detailed age categories; (iii) seasonal patterns of respiratory virus circulation and, where feasible, differences between northern and southern Vietnam; (iv) the distribution of single and multiple viral infections to identify the most common respiratory viruses causing single-infections. Comparisons of proportions were performed using the chi-square (χ^2^) test, with *p* < 0.05 considered statistically significant.

## 4. Results

### 4.1. Number of Samples Analyzed

Over the five-year period from 2020 to 2024, a total of 15,936 test results were analyzed. Among these, 9639 were from adults and 6297 from children. By year, the distribution was as follows: 1451 cases in 2020 (858 adults and 593 children), 1355 cases in 2021 (1174 adults and 181 children), 2890 cases in 2022 (2194 adults and 636 children), 4601 cases in 2023 (2405 adults and 2196 children), and 5639 cases in 2024 (3008 adults and 2631 children).

### 4.2. Respiratory Viruses Detected by the MPL Real-Time PCR Testing

Among 15,936 samples analyzed, 5081 were positive for respiratory viruses (31.88%). The yearly distribution of respiratory virus detection rates from 2020 to 2024 is presented in Table 2.

Over the 2020–2024 study period, the detection rate of respiratory viruses was consistently higher in children than in adults. The only exception occurred in 2021, when the detection rate in children (10.50%) was lower than in adults (15.76%). This year also exhibited the lowest overall respiratory viruses detection rate (15.06%) in the entire study population. Potential explanations for these variations are discussed in the following section.

Over the five-year period, MPL real-time PCR detected a total of 15 respiratory viruses, including InfA, InfB, InfC, Para1, Para2, Para3, ADV, RSV, Rhino, MeaVR, hMPV, Boca, hCoV, VZV, and CoV2. No detections were recorded for SARS-CoV or MERS-CoV, both of which had caused global outbreaks in previous decades. Similarly, RUBV was not detected in any specimens throughout the study period. Table 3 summarizes the detection frequencies of these 15 viruses identified by MPL real-time PCR from 2020 to 2024. The most frequently detected viruses were InfA, Para3, RSV, ADV, MeaVR, Rhino, hMPV, Boca and CoV2 whereas InfB, InfC, Para1, Para2, hCoV and VZV were detected less often. Because multiple viruses could be detected in a single specimen, the percentages in Table 2 do not sum to 100%.

**Figure 1:**
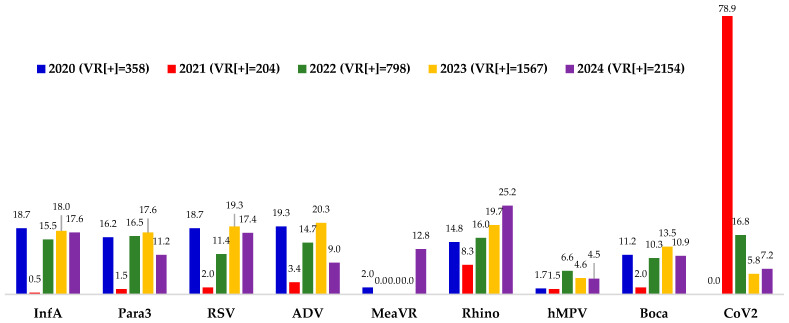
Detection rates (%) of nine common respiratory viruses from 2020 to 2024. Percentages represent the detection rate of each virus among respiratory virus-positive cases.

Figure 1 illustrates the yearly detection rates of the nine most prevalent respiratory viruses among the respiratory virus-positive cases. Several notable patterns were observed: (i) Regarding CoV2, no cases of CoV2 were detected in 2020 amidst the COVID-19 pandemic. In 2021, corresponding to the peak of COVID-19 pandemic in Vietnam, CoV2 accounted for 78.92% of detected respiratory viruses. In 2022, the post-pandemic year, CoV2 detection rate decreased to 16.79%, followed by further declines to 5.8% in 2023 and 7.2% in 2024. (ii) For MeaVR, the detection rate was only 2% in 2020, absent from 2021 to 2023, and increased markedly to 12.8% in 2024. (iii) Other common respiratory viruses were detected at notable rates in 2020, including InfA (15.54%), Para3 (16.54%), RSV (11.40%), ADV (14.66%), Rhino (16.04%), hMPV (6.64%) and Boca (10.28%). In 2021, detection rates of these viruses declined markedly. From 2022 to 2024, detection rates increased again, particularly for InfA (18.00% in 2023 and 17.55% in 2024), RSV (19.27% and 17.41%), and rhinovirus (19.66% and 25.16%), compared with 2022 levels.

### 4.3. Difference in Detection Rates of Respiratory Viruses Associated with Hospitalized Acute LRTI in Adults and Children

Table 4 presents the results and comparative analysis of respiratory viruses detected in hospitalized adults and children with acute LRTI. Pediatric patients were defined as those aged <16 years, while adults were ≥16 years of age. Several key findings can be summarized as follows: (i) The overall respiratory virus detection rate was higher in children than in adults. In particular, detection rates of Para1, Para3, RSV, ADV, MeaVR, Rhino, and Boca were significantly higher in children (*p* < 0.001). (ii) In contrast, InfA, InfB, CoV2 were detected more frequently in adults than in children, with differences also being statistically significant (*p* < 0.001). (iii) The detection rate of hMPV did not differ significantly between adults and children (*p* > 0.05). (iv) Detection rates of other respiratory viruses, including InfC, hCoV, Para2, and VZV, were very low in both groups, with no statistically significant differences (*p* > 0.05).

The respiratory virus detection rates across different age groups are summarized in Table 5. Pediatric patients were divided into four subgroups: <1 year (infants, breastfeeding period), 1–3 years (childcare age), 4–6 years (kindergarten age), and 7–15 years (primary and secondary school age). Adult patients were categorized into five subgroups: 16–40 years (young adults), 41–60 years (middle-aged adults), 61–70 years (early elderly), 71–85 years (elderly), and >85 years (very elderly).

Data from Table 5 can be summarized as follows: (i) CoV2 and InfA were detected at higher rates in adult age groups, with the highest prevalence observed in individuals aged ≥41 years; (ii) Para3 and RSV predominated among children under 3 years of age, particularly in those under 1 year old, where RSV was detected in 38.06% of cases; (iii) MeaVR and ADV were both common in pediatric patients but exhibited contrasting distribution patterns: MeaVR detection was highest in children under 1 year old (10.95%), whereas ADV was most prevalent among children aged 1–15 years (20.48% in those aged 1–3, 18.67% in 4–6, and 22.03% in 7–15 years); (iv) Although Rhino showed a significantly higher detection rate in children than in adults, age-specific analysis revealed the highest prevalence among children aged 1–6 years (27.31–27.43%). Detection rates remained relatively high and consistent among individuals aged 7–60 years (19.19–19.82%), with no notable differences among these age groups; (v) For Boca, the highest detection rate was observed in children aged 1–3 years (22.05%); (vi) hMPV showed similar and generally low detection rates across all age groups, with no substantial variation.

### 4.4. Seasonality of Respiratory Viruses

To avoid pandemic-related bias, only data from 2023 and 2024 were included in the seasonality analysis. Samples collected during these two years were further divided by geographic region: 9319 samples from the South of Vietnam and 921 samples from the North. Detailed results are presented in Table 6.

Analysis of the data presented in Table 5 revealed distinct seasonal patterns of respiratory virus circulation in Vietnam. In the South, the infection season for InfA extended from February to November, while Para3 predominated from November to April of the following year. RSV was most prevalent between May and November, and ADV between October and April. Rhino showed widespread circulation from September to July, with detection rates remaining relatively high even in August. hMPV infections occurred mainly from November to February, Boca from December to April, and CoV2 from April to June. For MeaVR, the measles outbreak that began in April 2024 resulted in a marked increase in detection rates (from 1.07% in April to 19.26% in December). In the North, seasonal trends were less distinct due to the smaller number of samples collected in 2023–2024. However, InfA infections were mainly detected between July and March, and ADV between May and October, while other respiratory viruses showed no clear seasonal distribution.

### 4.5. Single and Multiple Viral Infections in Respiratory Virus-Associated Hospitalized Acute LRTI Cases

Table 7 presents the number and proportion of single respiratory virus infections among the 5081 respiratory viruses-positive cases detected from 15,936 hospitalized acute LRTI cases during the five-year period (2020–2024). Of these, 4207 cases (82.8%) involved a single viral agent, while the remaining 17.2% showed multiple respiratory virus co-infections.

Analysis of the data in Table 6 showed that InfA, InfB, CoV2, and VZV had the highest proportions of single-agent infections, all exceeding 80%, with CoV2 being the highest at 88.54%. In contrast, Boca and hCoV exhibited the lowest single-agent detection rates, both below 45%. Para1, Para2, Para3, and ADV showed intermediate rates ranging from 55% to 60%, while InfC, Rhino, MeaVR, RSV, and hMPV had moderate single-agent detection rates between 63% and 77%.

## 5. Discussions

### 5.1. Diagnostic Limitations of Conventional Microbiological Methods

Once entering the lower respiratory tract, respiratory viruses invade and replicate within the epithelial cells of the respiratory mucosa, leading to cell destruction, virus-induced inflammation and lung tissue damage [36,37,38,39]. As a result, patients develop acute LRTI. Furthermore, respiratory viruses exhibit a high capacity for transmission through aerosols, respiratory droplets, and direct contact, contributing to the high prevalence of virus-associated respiratory infections [40,41,42]. In addition, by damaging the respiratory mucosa, respiratory viruses can facilitate secondary infections by other respiratory viruses, bacteria, or fungi, thereby worsening the severity of lower respiratory tract disease [43,44,45].

Since respiratory viruses are not part of the normal microbial flora of the upper respiratory tract [46,47,48], their detection in any respiratory specimen is generally considered sufficient to identify them as the causative agents of infection. However, because viruses are obligate intracellular pathogens, viral culture requires specialized facilities and is rarely feasible in routine clinical laboratories [49,50,51,52,53]. Consequently, conventional diagnosis relies on specific antigen detection or serological testing [49]. Nonetheless, serological testing is limited by the kinetics of the immune response. Determining active infection requires the specific detection of IgM antibodies, which are produced transiently during the acute immune response and decline after viral clearance [54,55]. For IgG, confirming an acute infection requires demonstrating a significant rise in titer between paired serum samples collected 10–14 days apart. Due to these diagnostic limitations and practical constraints, serological testing is now rarely used in clinical microbiology laboratories to confirm active respiratory viral infections. Between 2001 and 2005, a study conducted in Binh Thuan Province, central Vietnam, investigated viral causes of acute undifferentiated fever in 606 patients using serological testing for virus-specific IgA, IgG, and IgM antibodies [56]. Based on antibody kinetics indicative of recent infection, the detection rates were low, including human parainfluenza virus (4.7%), InfB virus (2.2%), InfA virus (1.9%), and RSV (0.6%). In contrast, seroprevalence reflecting past exposure, as determined by virus-specific IgG antibodies, was substantially higher: Para (56.8%), InfB (12.1%), InfA (5.9%), and RSV (6.8%). Direct detection of specific respiratory viral antigens in clinical specimens is primarily performed using two methods: direct fluorescent antibody (DFA) staining and immunochromatographic assays. DFA employs fluorochrome-labeled specific antibodies to visualize infected cells under a fluorescence microscope. Although this technique yields results within 1–2 h, it requires specialized equipment and interpretation skills. Furthermore, its sensitivity and specificity are variable, depending largely on antibody quality; consequently, it is rarely used in routine diagnostics. In contrast, immunochromatographic assays offer significant advantages: they can be performed at the point of care without specialized instrumentation and provide results rapidly, typically within 15–30 min [49,57]. These benefits have led to their widespread adoption for detecting respiratory viruses such as InfA, Para, ADV, RSV, and SARS-CoV-2 [49,57]. However, the sensitivity and specificity of these assays remain limited, preventing them from serving as the gold standard [58,59,60]. Moreover, they cannot detect the full spectrum of respiratory viruses potentially present in clinical specimens.

### 5.2. The Advantages of MPL Real-Time PCR Solution in Respiratory Virus Detection

Therefore, real-time PCR, which detects specific nucleic acid sequences of respiratory viruses in respiratory specimens, represents the most effective diagnostic approach. This technique has been applied for many years and has consistently demonstrated high efficiency in the detection of multiple respiratory viruses [26,61,62,63,64]. Several commercial MPL real-time PCR kits are now available and have been implemented in clinical microbiology laboratories in both automated and semi-automated formats. From a technological perspective, real-time PCR is also feasible for laboratories to develop independently due to the following advantages: (i) Specific primers and probes can be designed using dedicated software such as Primer Express 3.0.1, based on target gene sequences obtained from the NCBI GenBank database; and (ii) PCR reagents are affordable and widely available, in contrast to the expensive materials required for serology. Recognizing this, the WHO and CDC published specific primer and probe protocols for emerging viruses like SARS-CoV-2 to accelerate global testing implementation [65,66]. Using the primer and probe sequences recommended by the WHO and CDC for SARS-CoV-2 detection, we developed and implemented assays not only for identifying SARS-CoV-2, but also for other coronaviruses such as SARS-CoV, MERS-CoV, and hCoV [67,68]. Furthermore, we designed and applied assays capable of detecting SARS-CoV-2 variants, based on target-failure principle [69]. In 2018, we developed a multiplex real-time PCR platform capable of detecting a broad spectrum of respiratory pathogens, including bacteria, fungi, and viruses, as part of a national study coordinated by the Vietnam Lung Association [70]. This approach has also been applied in several studies in Vietnam [17,18,19,20,71]. However, those studies were limited in duration, the number of pathogens detected, or the patient groups involved. These limitations likely explain the differences between previous findings and the present study. To enable automated extraction of both DNA and RNA, we also developed an in-house reagent system using silica-coated magnetic beads, compatible with automated extraction instruments [72,73,74,75]. As a result, the workflow established in this study for respiratory viruses detection from respiratory specimens minimizes manual handling errors and delivers results within about three hours from test initiation. Although slightly slower than fully automated commercial systems, it remains a rapid and reliable diagnostic approach that can be readily applied in laboratories.

### 5.3. Overview of Respiratory Virus Detection Studies in Vietnam Using MPL Real-Time PCR

In Vietnam, several multiplex PCR studies have investigated respiratory viruses in patients with respiratory infections. For instance, from November 2004 to January 2008, a study at the Hospital for Tropical Diseases (Ho Chi Minh City) analyzed 309 hospitalized pediatric patients (under 15 years old). This study utilized a commercial multiplex kit (Seeplex™ RV Detection, Seegene) alongside additional monoplex real-time PCR assays to target 12 respiratory viruses [15]. The results showed that 222 cases (71.8%) were positive for at least one respiratory virus. Among these, RSV was the most common (73 cases, 23.6%), followed by InfA (8.7%), InfB (7.8%), Boca (16.2%), enterovirus (9.1%), hMPV (6.8%), Para (6.1%)-including Para1 (2.6%), Para2 (1.0%), and Para3 (2.6%), as well as coronavirus (7.8%), ADV (4.9%), and Rhino (3.6%). The study also analyzed respiratory viruses detection by age group and by single versus co-infections, making it an early and comprehensive investigation in this field. However, as the Hospital for Tropical Diseases is not a specialized pediatric center, the study population remained relatively small despite the three-year study period.

A study conducted at Khanh Hoa General Hospital from January 2007 to March 2008 examined 958 hospitalized pediatric patients (94% under five years old) using MPL PCR assay to detect 13 respiratory viruses. The results showed that 69% of cases were positive for respiratory viruses. The most prevalent respiratory viruses were Rhino (28%), RSV (23%), and InfA (15%), followed by ADV (5%), hMPV (5%), Para3 (4%), and Boca (2%). Detection rates of Para1, Para2, and InfB were low, each detected in only about 1.5% of cases [11]. At the same hospital, another study using the same MPL PCR was conducted from April 2007 to March 2010 on 397 children under five years old hospitalized with LRTIs. The study aimed to detect RSV and other respiratory viruses. Results showed that 60.9% of cases were positive for at least one respiratory viruses, with the most common pathogens being Rhino (27.7%), RSV (23.7%), and InfA (9.1%), followed by ADV (4.0%), hMPV (3.5%), Para3 (2.8%), Boca (1.8%), InfB (0.8%), Para1 (0.3%), and Para2 (0.5%) [12]. An interesting finding of this study was that, for each detected respiratory viruses, the detection rate was consistently lower among patients with LRTIs compared with those with upper respiratory infections. This observation is reasonable, as most respiratory viruses initially infect the upper respiratory tract before progressing to the lower tract. Therefore, nasopharyngeal or throat swabs remain valid specimens for detection of respiratory viruses.

A study conducted at Children’s Hospital 2 in Ho Chi Minh City from April 2010 to May 2011 investigated respiratory viruses in 1082 pediatric patients diagnosed with respiratory infection [14]. This study employed MPL PCR to detect 13 respiratory viruses, using a methodology similar to previously described studies at Khanh Hoa General Hospital [11,12]. The results showed that 64% of cases were positive for respiratory viruses, with 9.1% involving co-infections. Among the detected viruses, the most common were Rhino (30%), RSV (23.8%), and Boca (7.2%), followed by Para3 (5.3%), Para1 (3.3%), and InfA (3.2%).

A study conducted at the National Children’s Hospital on 194 infants aged 2–24 months hospitalized with LRTIs during a six-month period (November 2014-June 2015) employed MPL PCR based on the xTAG Respiratory Viral Panel (RVP) technique [76]. The study found that 143 cases (73.7%) were positive for respiratory viruses. Among these, 73 (37.6%) were RSV, 62 (32.0%) Rhino, 28 (14.4%) Para, 15 (7.7%) ADV, 9 (4.6%) Boca, 6 (3.1%) InfA/B, 3 (1.5%) hMPV, and 3 (1.5%) hCoV. The relatively high detection rate in this study, compared with our study, likely reflects the inclusion of infants and the timing of sampling during the rainy season [76].

A multicenter study was conducted across five sites, including Ba Vi (Hanoi), Hue, Khanh Hoa, Dak Lak, and Dong Thap, from November 2012 to June 2016. This study analyzed 4326 hospitalized patients with respiratory infections to identify respiratory viruses using MPL real-time PCR for 14 common pathogens, following previously described protocols [77]. Viral pathogens were detected in 64% of cases, with 14% showing co-infections. The high overall positivity rate likely reflects the study population, as 79% of participants were children under five years old and only 21% were adults or older children. The most frequently detected viruses were RSV (23%), Rhino (13%), InfA (11%), and Boca (7%). Notably, this study incorporated metagenomic analysis using next-generation sequencing (NGS) to examine viral subgroups such as RSV, InfA, and Boca [77].

At Vinmec Hospital in Hanoi, between January 2019 and December 2023, real-time PCR assays using Allplex^TM^ respiratory panel 1 to 4 (Seegene) for detection respiratory pathogens were performed on 42,041 samples from 28,178 patients [61]. The results for detection respiratory viruses showed that 6614 (15.7%) were positive for InfA, 1677 (4.0%) for InfB, 810 (1.9%) for RSV, 413 (1.0%) for ADV, 161 (0.4%) for enterovirus, and 121 (0.3%) for hMPV. Epidemiological analysis revealed that the mean age of patients positive for ADV, enterovirus, or hMPV was approximately three years. In contrast, RSV-positive patients averaged one year of age, while InfA/InfB-positive patients averaged around five years. Regarding seasonality, the study noted that the most distinct seasonal pattern was observed for InfA, with peaks occurring during the winter and spring months [16].

### 5.4. Hospitalized Respiratory Infection Focus Reveals Distinct Age-Specific Respiratory Virus Patterns

Previous studies conducted in Vietnam have reported detection rates of respiratory viruses among hospitalized pediatric patients ranging from 60% to 70% when using MPL PCR or MPL real-time PCR. These rates were slightly higher than that observed in our study (52.98%). This difference may be due to variations in the study population, as our analysis primarily included patients hospitalized with acute LRTI, rather than the broader group of ARI, which also includes upper respiratory tract infections. The viruses with higher detection rates represent common respiratory viruses and are frequently associated acute LRTI requiring hospitalization. Regarding the distribution of respiratory viruses, most studies detected RSV as the predominant pathogen, usually exceeding 20%, as the study populations primarily consisted of children under five years of age. Our study showed a comparable finding, with RSV detected in 21.97% of pediatric cases and at a particularly high rate among children under one year of age (38.06%). In contrast, the study conducted at the Hospital for Tropical Diseases reported a lower RSV detection rate (8.7%) [15]. This finding is likely because the study population included children up to 15 years old, which reduced the overall rate when age groups were not analyzed separately.

Detection rates of other respiratory viruses varied among studies, likely reflecting differences in geographic location and study period. Two investigations provided extended epidemiological data over multiple years. The first was conducted in Nha Trang from January 2007 to April 2012 on 3431 hospitalized children with respiratory infections [13]. The second was a multicenter study conducted from November 2012 to June 2016 on 4326 patients, of whom 79% were under five years old [77]. However, neither study included detailed analyses of age-specific or seasonal distribution of respiratory viruses as presented here. Notably, regarding the re-emergence of measles in 2024, a clear seasonal pattern could not yet be established because this observation covered only a short period. Ongoing monitoring in subsequent years will be required, and control is expected once nationwide vaccination coverage is fully restored.

For adult patients, few studies in Vietnam have examined respiratory viruses in hospitalized cases, possibly because these viruses are often considered co-infecting agents with bacterial pathogens [78]. In the present dataset, respiratory viruses were also detected among adults, with an overall rate of 18.10%. InfA and CoV2 were the most frequent respiratory viruses, accounting for 28.28% and 25.62%, respectively, higher than the rates observed in children (10.22% and 2.82%).

### 5.5. Impact of the COVID-19 Pandemic and Age-Related Susceptibility Patterns

The study period spanned from 2020 to 2024, during which the years 2020–2022 were affected by the COVID-19 pandemic: 2020 marking its onset, 2021 its peak, and 2022 the post-outbreak period when residual effects persisted.

In 2020, although COVID-19 had been reported in many countries worldwide, only a few cases were recorded in Vietnam. Infected individuals were isolated in designated facilities rather than hospitalized in respiratory wards; therefore, no CoV2 associated cases of hospitalization for acute LRTI were documented. During this period, mask-wearing in public spaces was nearly universal due to national policy. However, the detection rates of other respiratory viruses remained relatively unchanged. This suggests that public masking mandates alone were insufficient to significantly reduce transmission in the absence of broader restrictions, likely due to confounding factors such as continued household transmission and indoor gatherings which were not restricted during this period.

In 2021, the detection rate of CoV2 reached 78.92%, while the rates for other viruses were markedly lower. This pattern coincided with the COVID-19 pandemic in Vietnam. These findings indicate that in a viral pandemic, a single causative agent tends to dominate hospitalized respiratory cases, while a rise in detections without such dominance is better classified as an outbreak. Consequently, data from 2021 represents a unique epidemiological outlier and should be interpreted with caution when assessing long-term viral circulation trends.

In the study, with the analysis data from Table 3, the detection rates of the respiratory viruses Para1, Para3, RSV, ADV, MeaVR, Rhino, and Boca in children were significantly higher than in adults (*p* < 0.001). This difference is likely because many adults were previously infected with these viruses during childhood and have developed long-term protective immunity. For instance, most adults have experienced MeaVR infection early in life, and a single infection confers lifelong immunity. However, the detection rates of InfA, InfB, CoV2 in adults were significantly higher than in children (*p* < 0.001). One possible explanation is that these viruses exhibit antigenic variation (particularly InfA and CoV2), enabling partial escape from preexisting immunity, especially in the elderly adults who often have multiple risk factors and comorbidities such as obesity, cardiovascular conditions, and diabetes. Furthermore, while clinical comorbidities were not analyzed in this study, established literature suggests that age-related risk factors and waning immunity likely contribute to the increased severity and hospitalization rates observed in this demographic.

## 6. Conclusions

In Vietnam, commercial real-time PCR and multiplex real-time PCR (MPL real-time PCR) assays have been adopted in several hospitals for the detection of respiratory viruses in hospitalized patients, particularly in pediatric settings. However, their widespread use remains limited by high cost and restricted target coverage, as most commercial kits include only a limited number of common pathogens and often exclude emerging viruses.

To address these limitations, the Vietnam Research and Development Institute of Clinical Microbiology developed an in-house MPL real-time PCR system capable of detecting a broad range of respiratory viruses, including emerging pathogens such as measles virus and SARS-CoV-2. This system has been applied in multiple studies in Vietnam, including during the COVID-19 pandemic [11,12,13,14,46,47,48,49,50], and has been adopted for routine use in appropriately equipped clinical microbiology laboratories due to its reliability and cost-effectiveness.

Using this system, 15,936 respiratory specimens collected from hospitalized patients across Vietnam between 2020 and 2024 were analyzed, providing valuable epidemiological insights into respiratory viruses in both pediatric and adult populations. However, the study is limited by its retrospective design, the absence of clinical severity and outcome data, the exclusive focus on viral pathogens without assessment of bacterial co-infections. Furthermore, seasonality analyses were constrained by uneven geographical representation, with a significantly larger sample size from the South compared to the North. In addition, enterovirus was not included as a target despite its low reported prevalence in previous Vietnamese studies. Despite these constraints, the study underscores the importance of accessible molecular testing. Ongoing surveillance is therefore needed to further characterize the epidemiology of emerging respiratory viruses.

**Supplementary Method:** Sputum samples were homogenized with freshly prepared 1% N-acetyl-L-cysteine (NALC) in TE 1× buffer by mixing equal volumes of sample and NALC, followed by thorough vortexing. Other sample types were briefly vortexed to ensure homogeneity without NALC. Total nucleic acids (DNA and RNA) were extracted using magnetic bead-based technology following cell lysis. Purified nucleic acids were eluted in 1× TE buffer and stored until subsequent MPL real-time PCR testing.

The MPL real-time PCR was performed by adding extracted nucleic acid from each specimen into a PCR tube containing MPL real-time PCR master mix prepared by the Viet Nam Research and Development Institute of Clinical Microbiology (VCM). Each master mix contained specific primers and TaqMan probes targeting four distinct nucleic acid sequences. The specific primers and probes sequences are shown in Table 1. The thermal cycling protocol consisted of reverse transcription at 45 °C for 10 min (for RNA targets); initial denaturation and hot-start polymerase activation at 95 °C for 15 min; followed by 40 amplification cycles, each comprising 2 steps: denaturation at 95 °C for 15 s and annealing at 60 °C for 1 min. Fluorescence signals were recorded during the annealing step using four detection channels—FAM, HEX, Texas Red, and Cy5—corresponding to the fluorophores of the specific TaqMan probes. A respiratory viral target was considered positive when the cycle threshold (Ct) value was below 38.5, as recommended by the manufacturer.

## Figures and Tables

**Table 1:** The primers and probes used in the MPL real-time PCR testing for detection of respiratory viruses.

**Influenza A virus**. Terrier O et al. 2011. [21]		**Influenza B virus**. van Elden LJ et al. 2001 [22]	
FLUA-F	CTTCTAACCGAGGTCGAAACGTA	FLUBHA-F	AAATACGGTGGATTAAACAAAAGCAA
FLUA-R	GGTGACAGGATTGGTCTTGTCTTTA	FLUBHA-R	CCAGCAATAGCTCCGAAGAAA
FLUA-P	TCAGGCCCCCTCAAAGCCGAG	FLUBHA-P	CACCCATATTGGGCAATTTCCTATGGC
**Influenza C virus**. WHO 2020 [23]		**Parainfluenza 1 virus** Templeton KE et al. 2004 [24]	
FluC-Fedit *	GCTTTGGACTTGCTTATGAAGACTTC	Para1-Fedit *	GCCAACCTACAAGGCAACAAC
FluC-Redit *	TCACCGACTCTGAAGTTTCCTATTT	Para1-Redit *	CTTCCTGCTGGTGTGTTAATGTG
FluC-Pedit*	AGCCACCCTCTTAAGTTGAGAAACAGAATG	Para1-Pedit *	TGGTCTACAACCCGAAATGATAACTCCACG
**Parainfluenza 2 virus** Templeton KE et al. 2004 [24]		**Parainfluenza 3 virus** Templeton KE et al. 2004 [24]	
Para2-Fedit *	TGAAACCATTTACCTAAGTGATGGAA	Para3-Fedit *	CAGGGAGCATTGTGTCATCTGT
Para2-Redit *	CGTGGCATAATCTTCTTTTTCAGA	Para3-Redit *	GTGTGTAATGCAGCTCGTTGTTG
Para2-Pedit *	CAATCGCAAAAGCTGTTCAGTCACTGCT	Para3-Pedit *	CTGAAAGGGTAAACGAGCTGGCCATCC
**Respiratory syncytial virus**. Jokela et al. 2010 [25]		**Human rhinovirus**. Gunson RN et al. 2005 [26]	
RSV-Fedit *	GAAACATACGTGAACAARCTTCA	HRV-Fedit *	GATTGGACAGGGTGTGAAGAGC
RSV-Redit *	GCACCCATATTGTWAGTGATGC	HRV-Redit *	CCAAAGTAGTCGGTCCCATCC
RSV-P	CGAAGGCTCCACATACACAGCWGCTGT	HRV-Pedit *	CCTCCGGCCCCTGAATGCG
**Human adenovirus**. Lieberman D et al. 2009 [27]		**Human metapneumovirus**. Mackay IM et al. 2003 [28]	
Adeno-Fedit *	CCCAGTGGTCTTACATGCACAT	hMPV-Fedit *	CTAACCGTGTACTAAGTGATGCACTCA
Adeno-Redit *	CCACGGTGGGGTTTCTAAACT	hMPV-Redit *	GCTTTGCCATACTCAATGAACAAAC
Adeno-Pedit *	TCGGAGTACCTGAGCCCGGGTCTG	hMPV-Pedit *	ACCCTAGAATGGACATCCCAAAAATTGCC
**Human bocavirus**. Lu X et al. 2006 [29]		**Measles virus**. Pham VH et al. 2024 [30]	
Boca-F	AGAGGCTCGGGCTCATATCA	MeasVR-Fedit *	CCTTATTTGTGGAGTCTCCAGGTC
Boca-R	CACTTGGTCTGAGGTCTTCGAA	MeasVR-Redit *	GCCTCATCCTCCATGTTGGTAC
Boca-Pedit *	AGGAACACCCAATCARCCACCTATCGT	MeasVR-Pedit *	TCAGAGGATCACCGATGACCCTGACG
**Varicella-Zoster virus**. Weidmann M et al. 2003 [31]		**Rubella virus**. Revello, M. G. et al. 1997 [32]	
VZV-F	CGGCATGGCCCGTCTAT	Rubella-Fedit *	CTCGAGGTCCAGGTCCTGC
VZV-R	TCGCGTGCTGCGGC	Rubella-Redit *	GAATGGCGTTGGCAAACC
VZV-P	ATTCAGCAATGGAAACACACGACGCC	Rubella-Pnew *	TGAACCACACCGGCAATCAGCAG
**Human coronavirus**. Gunson RN et al. 2005 [26]		**MERS-CoV**. Corman VM et al. 2012 [33]	
HCoV-F	CAGTCAAATGGGCTGATGCA	upE-TqF	GCAACGCGCGATTCAGTT
HCoV-R	AAAGGGCTATAAAGAGAATAAGGTATTCT	upE-tqR	GCCTCTACACGGGACCCATA
HCoV-P	CCCTGACGACCACGTTGTGGTTCA	upE-TqPR	CTCTTCACATAATCGCCCCGAGCTCG
**SARS-CoV**. Briese T et al. 2025 [34]		**SARS-CoV-2** Novel Coronavirus (2019-nCoV) [35]	
CoSAR_TqF	AAGCCTCGCCAAAAACGTAC	CoV2-F	TTACAAACATTGGCCGCAAA
CoSAR_TqR	AAGTCAGCCATGTTCCCGAA	CoV2-R	GCGCGACATTCCGAAGAA
CoSAR_TqPR	TCACGCATTGGCATGGAAGTCACAC	CoV2-P	ACAATTTGCCCCCAGCGCTTCAG

Fedit *, Redit *, Pedit * and Pnew * were edited by VCM from the references to obtain the suitable annealing temperature (#60 °C for F and R, and #70 °C for P).

**Table 2:** Annual number of samples and detection rates of respiratory viruses from 2020 to 2024.

Year	Number of Samples			% Respiratory Viruses Detected		
				Adults and Children	Adults	Children
	Total	Adults	Children	% (n)	% (n)	% (n)
2020	1451	858	593	24.67 (358)	12.47 (107)	42.33 (251)
2021	1355	1174	181	15.06 (204)	15.76 (185)	10.50 (19)
2022	2890	2194	696	27.61 (798)	20.01 (439)	51.58 (359)
2023	4601	2405	2196	34.06 (1567)	16.42 (395)	53.37 (1172)
2024	5639	3008	2631	38.20 (2154)	20.58 (619)	58.34 (1535)
Total	15,936	9639	6297	31.88 (5081)	18.10 (1745)	52.98 (3336)

Adults: ≥16 years; Children: <16 years.

**Table 3:** Number of cases and percentage of respiratory viruses associated with acute LRTI from 2020 to 2024.

Respiratory Viruses	2020 N (%)	2021 N (%)	2022 N (%)	2023 N (%)	2024 N (%)	2020–2024 N (%)
**InfA**	67 (18.72)	1 (0.49)	124 (15.54)	282 (18.00)	378 (17.55)	852 (16.77)
InfB	2 (0.56)	0 (0.00)	79 (9.90)	2 (0.13)	55 (2.55)	138 (2.72)
InfC	6 (1.68)	3 (1.47)	8 (1.00)	11 (0.70)	2 (0.09)	30 (0.59)
Para1	13 (3.63)	2 (0.98)	4 (0.50)	27 (1.72)	29 (1.35)	75 (1.48)
Para2	6 (1.68)	0 (0.00)	1 (0.13)	15 (0.96)	25 (1.16)	47 (0.93)
**Para3**	58 (16.20)	3 (1.47)	132 (16.54)	275 (17.55)	241 (11.19)	709 (13.95)
**RSV**	67 (18.72)	4 (1.96)	91 (11.40)	302 (19.27)	375 (17.41)	839 (16.51)
**ADV**	69 (19.27)	7 (3.43)	117 (14.66)	318 (20.29)	193 (8.96)	704 (13.86)
**MeaVR**	7 (1.96)	0 (0.00)	0 (0.00)	0 (0.00)	276 (12.81)	283 (5.57)
**Rhino**	53 (14.80)	17 (8.33)	128 (16.04)	308 (19.66)	542 (25.16)	1048 (20.63)
**hMPV**	6 (1.68)	3 (1.47)	53 (6.64)	72 (4.59)	96 (4.46)	230 (4.53)
**Boca**	40 (11.17)	4 (1.96)	82 (10.28)	212 (13.53)	235 (10.91)	573 (11.28)
hCoV	3 (0.84)	1 (0.49)	0 (0.00)	4 (0.26)	4 (0.19)	12 (0.24)
**CoV2**	0 (0.00)	161 (78.92)	134 (16.79)	91 (5.81)	155 (7.20)	541 (10.65)
VZV	3 (0.84)	0 (0.00)	0 (0.00)	0 (0.00)	7 (0.32)	10 (0.20)

Respiratory viruses shown in bold had higher detection frequencies. Values in parentheses indicate the percentage of each virus among respiratory virus-positive cases.

**Table 4:** Number and percentage of respiratory viruses detected in hospitalized acute LRTI cases among adults and children. *p*-values were calculated using the chi-square test; *p* < 0.05 was considered statistically significant.

Respiratory Virus	Children N (%)	Adults N (%)	χ^2^	*p*
InfA	341 (10.22)	511 (29.28)	298.3	<0.001
InfB	52 (1.56)	86 (4.93)	49.23	<0.001
InfC	19 (0.57)	11 (0.63)	0.07	>0.05
Para1	65 (1.95)	10 (0.57)	14.9	<0.001
Para2	38 (1.14)	9 (0.52)	4.86	>0.05
Para3	561 (16.82)	148 (8.84)	66.29	<0.001
RSV	733 (21.97)	106 (6.07)	210.05	<0.001
ADV	594 (17.81)	110 (6.30)	126.99	<0.001
MeaVR	278 (8.33)	5 (0.29)	141.05	<0.001
Rhino	775 (23.23)	273 (15.64)	40.28	<0.001
hMPV	153 (4.59)	77 (4.41)	0.08	>0.05
Boca	542 (16.25)	31 (1.78)	239.77	<0.001
hCoV	9 (0.27)	3 (0.17)	0.143*	>0.05
CoV2	94 (2.82)	447 (25.62)	625.93	<0.001
VZV	3 (0.09)	7 (0.40)	4.176*	>0.05
Respiratory virus [+]	3336 (52.98)	1745 (18.10)	2132.9	<0.001

Note: *p*-values were calculated using the chi-square test. Statistical significance was defined as *p* < 0.05.

**Table 5:** Respiratory virus detection rates across age groups.

	InfA	Para3	RSV	ADV	MeaVR	Rhino	hMPV	Boca	CoV2	VR[+]
<1 Y	6.57	**18.87**	**38.06**	11.68	**10.95**	17.94	4.69	10.74	2.71	**52.78**
1–3 Y	9.67	**18.13**	**16.56**	**20.48**	**6.16**	**27.31**	4.53	**22.05**	2.24	**63.12**
4–6 Y	20.25	10.13	8.44	**18.57**	**8.02**	**27.43**	5.49	9.70	4.22	**41.29**
7–15 Y	14.54	9.25	6.61	**22.03**	**9.69**	**19.82**	4.85	7.05	7.93	25.17
16–40 Y	**22.67**	9.88	6.98	9.88	0.58	**19.19**	1.16	4.65	**16.28**	19.77
41–60 Y	**24.77**	10.53	3.10	8.67	0.62	**19.81**	5.57	3.10	**24.15**	18.67
61–70 Y	**32.77**	7.47	5.78	4.82	0.48	12.29	3.61	0.48	**29.16**	19.59
71–85 Y	**32.21**	8.54	8.19	4.80	0.00	14.77	5.87	1.25	**24.56**	17.33
>85 Y	**27.85**	6.75	5.06	6.75	0.00	16.46	3.38	0.84	**29.96**	15.84
Children	10.22	**16.82**	**21.97**	**17.81**	**8.33**	**23.23**	4.59	**16.25**	2.82	**52.98**
Adults	**29.28**	8.48	6.07	6.30	0.29	15.64	4.41	1.78	**25.62**	18.10
Overall	16.77	**13.95**	**16.51**	**13.86**	**5.57**	**20.63**	4.53	**11.28**	10.65	**31.88**

Bolded values indicate higher detection rates within each age group.

**Table 6:** Monthly detection rates (%) of respiratory viruses in 2023 and 2024 based on 9319 samples from the South and 921 samples from the North of Vietnam.

	InfA	Para3	RSV	ADV	MeaVR	Rhino	hMPV	Boca	CoV2	VR[+]
	Results from 9319 samples in the South of Vietnam									
Jan	4.21	**25.89**	2.59	**25.89**	0.00	**36.25**	**5.50**	**20.39**	5.83	**44.02**
Feb	**15.54**	**23.90**	5.18	**19.12**	0.00	**37.85**	**5.58**	**13.94**	2.39	**38.20**
Mar	**15.14**	**23.11**	6.37	**15.94**	0.00	**36.65**	2.79	**12.75**	2.39	34.86
Apr	**14.97**	**15.51**	**13.90**	**12.83**	1.07	**19.25**	4.81	**18.18**	**11.76**	30.41
May	**9.71**	8.74	**25.73**	10.19	2.43	**19.42**	0.00	9.71	**29.13**	29.30
Jun	**16.30**	8.70	**30.98**	3.80	4.35	**15.22**	3.26	11.96	**19.57**	28.66
Jul	**22.62**	5.43	**35.75**	5.43	4.98	**24.89**	1.36	9.95	6.79	30.27
Aug	**33.18**	2.84	**29.86**	9.48	**9.95**	9.95	0.95	9.48	5.21	30.49
Sep	**18.14**	6.51	**35.35**	5.58	**10.70**	**24.65**	2.33	9.77	0.47	33.75
Oct	**25.25**	4.70	**32.67**	**11.88**	**7.92**	**16.09**	4.95	6.44	1.24	**42.26**
Nov	**24.14**	**11.36**	**16.84**	**14.00**	**13.59**	**14.81**	**7.10**	9.94	2.23	**44.45**
Dec	8.89	**22.59**	8.70	**17.96**	**19.26**	**22.41**	**7.96**	**15.19**	4.26	**46.71**
	Results from 921 samples in the North of Vietnam									
Jan	**37.50**	**12.50**	0.00	6.25	0.00	**25.00**	**6.25**	0.00	**12.50**	34.78
Feb	**26.67**	6.67	**13.33**	0.00	0.00	**26.67**	**13.33**	0.00	6.67	22.39
Mar	**16.00**	**12.00**	**12.00**	8.00	0.00	**52.00**	**12.00**	4.00	0.00	37.88
Apr	12.50	**16.67**	**12.50**	4.17	0.00	**16.67**	0.00	**16.67**	**33.33**	28.24
May	11.76	**11.76**	**23.53**	**23.53**	5.88	0.00	0.00	**23.53**	**11.76**	19.54
Jun	9.52	**9.52**	**19.05**	**23.81**	0.00	**28.57**	4.76	4.76	**9.52**	25.30
Jul	**20.00**	6.67	3.33	**13.33**	0.00	**16.67**	0.00	**13.33**	**30.00**	33.71
Aug	**35.00**	0.00	0.00	**40.00**	0.00	**10.00**	0.00	**15.00**	**25.00**	20.62
Sep	**20.00**	**20.00**	**13.33**	**26.67**	0.00	6.67	0.00	6.67	**20.00**	20.83
Oct	**21.43**	7.14	**14.29**	**21.43**	0.00	**35.71**	0.00	7.14	0.00	20.29
Nov	**44.44**	**11.11**	0.00	0.00	**11.11**	**27.78**	0.00	5.56	0.00	27.27
Dec	**47.06**	**11.76**	8.82	2.94	0.00	**29.41**	0.00	2.94	0.00	36.17

Bolded values indicate higher detection rates within each month.

**Table 7:** Cases and percentages of single viral infections among respiratory viruses detected between 2020 and 2024.

	2020 to 2024		
	Detected Cases	Single-Infection Cases	%Single Infection Among Detected Cases
InfA	852	717	**84.15**
InfB	138	115	**83.33**
InfC	30	19	63.33
Para1	75	41	54.67
Para2	47	27	57.45
Para3	709	397	55.99
RSV	839	633	75.45
ADV	704	417	59.23
MeaVR	283	200	70.67
Rhino	1048	723	68.99
hMPV	230	177	76.96
Boca	573	249	43.46
hCoV	12	5	41.67
CoV2	541	479	**88.54**
VZV	10	8	**80.00**
Respiratory virus	5081	4207	82.80

Bolded values indicate respiratory viruses with the highest proportions of single-agent infection

## Data Availability

The datasets generated and analyzed during the current study are available from the corresponding authors on reasonable request.

## Abbreviations

The following abbreviations are used in this manuscript:

LRTI	lower respiratory tract infection
PCR	polymerase chain reaction
rPCR	real-time PCR
InfA	influenza A
InfB	influenza B
InfC	influenza C
Para	parainfluenza virus
Para1-3	parainfluenza viruses 1-3
RSV	respiratory syncytial virus
ADV	human adenovirus
MeaVR	measles virus
Rhino	rhinovirus
hMPV	human metapneumovirus
Boca	bocavirus
hCoV	human coronavirus HKU1
CoV2	SARS-CoV-2
SARS	SARS-CoV
MERS	MERS-CoV
VZV	varicella-zoster virus
RUBV	rubella virus

## References

[1] Tang Z., Fan H., Tian Y., Lv Q. (2025). Epidemiological characteristics of six common respiratory pathogen infections in children. Microbiol. Spectr..

[2] Akkoc G., Dogan C., Bayraktar S., Sahin K., Elevli M. (2021). Evaluation of viral respiratory pathogens in children aged under five hospitalized with lower respiratory tract infections. North. Clin. Istanb..

[3] Burk M., El-Kersh K., Saad M., Wiemken T., Ramirez J., Cavallazzi R. (2016). Viral infection in community-acquired pneumonia: A systematic review and meta-analysis. Eur. Respir. Rev..

[4] Ren L., Xiang Z., Guo L., Wang J. (2012). Viral infections of the lower respiratory tract. Curr. Infect. Dis. Rep..

[5] Pavia A.T. (2011). Viral infections of the lower respiratory tract: Old viruses, new viruses, and the role of diagnosis. Clin. Infect. Dis..

[6] Tchatchouang S., Kenmoe S., Nzouankeu A., Njankouo-Ripa M., Penlap V., Donkeng V., Pefura-Yone E., Fonkoua M., Eyangoh S., Njouom R. (2023). Viral etiology of lower respiratory tract infections in adults in the pre-COVID-19 pandemic era: A cross-sectional study in a single center experience from Cameroon. Health Sci. Rep..

[7] Weile J., Knabbe C. (2009). Current applications and future trends of molecular diagnostics in clinical bacteriology. Anal. Bioanal. Chem..

[8] Mothershed E.A., Whitney A.M. (2006). Nucleic acid-based methods for the detection of bacterial pathogens: Present and future considerations for the clinical laboratory. Clin. Chim. Acta.

[9] Prasad S., Lownik E., Ricco J. (2016). Viral Infections of the Respiratory Tract. Fam. Med..

[10] Kuchar E., Miśkiewicz K., Nitsch-Osuch A., Szenborn L. (2015). Pathophysiology of Clinical Symptoms in Acute Viral Respiratory Tract Infections. Adv. Exp. Med. Biol..

[11] Yoshida L.M., Suzuki M., Yamamoto T., Nguyen H.A., Nguyen C.D., Nguyen A.T., Oishi K., Vu T.D., Le T.H., Le M.Q. (2010). Viral pathogens associated with acute respiratory infections in central vietnamese children. Pediatr. Infect. Dis. J..

[12] Yoshida L.-M., Suzuki M., Nguyen H.A., Le M.N., Vu T.D., Yoshino H., Schmidt W.-P., Nguyen T.T.A., Le H.T., Morimoto K. (2013). Respiratory syncytial virus: Co-infection and paediatric lower respiratory tract infections. Eur. Respir. J..

[13] Althouse B.M., Flasche S., Minh L.N., Thiem V.D., Hashizume M., Ariyoshi K., Anh D.D., Rodgers G.L., Klugman K.P., Hu H. (2018). Seasonality of respiratory viruses causing hospitalizations for acute respiratory infections in children in Nha Trang, Vietnam. Int. J. Infect. Dis..

[14] Tran D.N., Trinh Q.D., Pham N.T.K., Vu M.P., Ha M.T., Nguyen T.Q.N., Okitsu S., Hayakawa S., Mizuguchi M., Ushijima H. (2016). Clinical and epidemiological characteristics of acute respiratory virus infections in Vietnamese children. Epidemiol. Infect..

[15] Do A.H.L., Van Doorn H.R., Nghiem M.N., E Bryant J., Hoang T.H.T., Do Q.H., Le Van T., Tran T.T., Wills B., Van Nguyen V.C. (2011). Viral Etiologies of Acute Respiratory Infections among Hospitalized Vietnamese Children in Ho Chi Minh City, 2004–2008. PLoS ONE.

[16] Ho N.T., Nguyen H.t.T.T., Hoang H.T., Pham D.V., Nguyen Q.N., Le H.T.M., Pham A.N., Doan P.M. (2025). Emerging pathogens associated with acute respiratory infections in children in Hanoi, Vietnam: An analysis of microbiology assay data from 2019 to 2023. F1000Research.

[17] Quang K.T., Do H.T., Hung V.P., Vu T.N., Xuan B.T., Larsson M., Duong-Quy S., Nguyen-Thi-Dieu T. (2022). Study on the co-infection of children with severe community-acquired pneumonia. Pediatr. Int..

[18] Ly V.K., Pham V.H., Van Ly X., Pham P.M. (2024). Bacterial and viral co-infections in community-acquired pneumonia in adults: A prospective study of multiple hospital centers. MedPharmRes.

[19] Tran K.Q., Pham V.H., Vo C.M., Pham Q.M., Nguyen P.M. (2024). Comparison of Real-time Polymerase Chain Reaction and Culture for Targeting Pathogens in Pediatric Severe Community-Acquired Pneumonia. Turk. Arch. Pediatr..

[20] Nguyen S.T.T., Tran T.A., Vo G.V. (2024). Severe Pneumonia Caused by Respiratory Syncytial Virus and Adenovirus in Children from 2 to 24 Months at Children’s Hospital 1 in Ho Chi Minh City, Vietnam. Viruses.

[21] Terrier O., Josset L., Textoris J., Marcel V., Cartet G., Ferraris O., N’guyen C., Lina B., Diaz J.-J., Bourdon J.-C. (2011). Cellular transcriptional profiling in human lung epithelial cells infected by different subtypes of influenza A viruses reveals an overall down-regulation of the host p53 pathway. Virol. J..

[22] van Elden L.J., Nijhuis M., Schipper P., Schuuman R., van Loon A.M. (2001). Simultaneous detection of influenza viruses A and B using real-time quantitative PCR. J. Clin. Microbiol..

[23] World Health Organization (2020). WHO Information for the Molecular Detection of Influenza Viruses.

[24] Templeton K.E., Scheltinga S.A., Beersma M.F.C., Kroes A.C.M., Claas E.C.J. (2004). Rapid and sensitive method using multiplex real-time PCR for diagnosis of infections by influenza a and influenza B viruses, respiratory syncytial virus, and parainfluenza viruses 1, 2, 3, and 4. J. Clin. Microbiol..

[25] Jokela P., Piiparinen H., Luiro K., Lappalainen M. (2010). Detection of human metapneumovirus and respiratory syncytial virus by duplex real-time RT-PCR assay in comparison with direct fluorescent assay. Virology.

[26] Gunson R.N., Collins T.C., Carman W.F. (2005). Real-time RT-PCR detection of 12 respiratory viral infections in four triplex reactions. J. Clin. Virol..

[27] Lieberman D., Lieberman D., Shimoni A., Keren-Naus A., Steinberg R., Shemer-Avni Y. (2009). Identification of respiratory viruses in adults: Nasopharyngeal versus oropharyngeal sampling. J. Clin. Microbiol..

[28] Mackay I.M., Jacob K.C., Woolhouse D., Waller K., Syrmis M.W., Whiley D.M., Siebert D.J., Nissen M., Sloots T.P. (2003). Molecular assays for detection of human metapneumovirus. J. Clin. Microbiol..

[29] Lu X., Chittaganpitch M., Olsen S.J., Mackay I.M., Sloots T.P., Fry A.M., Erdman D.D. (2006). Real-Time PCR Assays for Detection of Bocavirus in Human Specimens. J. Clin. Microbiol..

[30] Pham V.H., Nguyet D.P.H., Mai K.N.H., Truong K.H., Huynh L.V., Pham T.H., Abe K. (2024). Measles Epidemics Among Children in Vietnam: Genomic Characterization of Virus Responsible for Measles Outbreak in Ho Chi Minh City. EBioMedicine.

[31] Weidmann M., Meyer-König U., Hufert F.T. (2003). Rapid Detection of Herpes Simplex Virus and Varicella-Zoster Virus Infections by Real-Time PCR. J. Clin. Microbiol..

[32] Revello M.G., Baldanti F., Sarasini A., Zavattoni M., Torsellini M., Gerna G. (1997). Prenatal diagnosis of rubella virus infection by direct detection and semiquantitation of viral RNA in clinical samples by reverse transcription-PCR. J. Clin. Microbiol..

[33] Corman V.M., Eckerle I., Bleicker T., Zaki A., Landt O., Eschbach-Bludau M., van Boheemen S., Gopal R., Ballhause M., Bestebroer T.M. (2012). Detection of a novel human coronavirus by real-time reverse-transcription polymerase chain reaction. Eurosurveillance.

[34] Briese T., Palacios G., Kokoris M., Jabado O., Liu Z., Renwick N., Kapoor V., Casas I., Pozo F., Limberger R. (2005). Diagnostic system for rapid and sensitive differential detection of pathogens. Emerg. Infect. Dis..

[35] Center for Diseases and Control Prevention (2020). CDC 2019 Novel Coronavirus (2019-nCoV) Real-Time RT-PCR Panel Primers and Probes.

[36] Troy N.M., Bosco A. (2016). Respiratory viral infections and host responses; insights from genomics. Respir. Res..

[37] Nainwal N. (2022). Treatment of respiratory viral infections through inhalation therapeutics: Challenges and opportunities. Pulm. Pharmacol. Ther..

[38] Payne S. (2023). Virus interactions with the cell. Viruses.

[39] Spacova I., De Boeck I., Bron P.A., Delputte P., Lebeer S. (2021). Topical Microbial Therapeutics against Respiratory Viral Infections. Trends Mol. Med..

[40] Leung N.H.L. (2021). Transmissibility and transmission of respiratory viruses. Nat. Rev. Microbiol..

[41] Kutter J.S., Spronken M.I., Fraaij P.L., Fouchier R.A., Herfst S. (2018). Transmission routes of respiratory viruses among humans. Curr. Opin. Virol..

[42] Shiu E.Y., Leung N.H., Cowling B.J. (2019). Controversy around airborne versus droplet transmission of respiratory viruses: Implication for infection prevention. Curr. Opin. Infect. Dis..

[43] Liu Y., Ling L., Wong S.H., Wang M.H., Fitzgerald J., Zou X., Fang S., Liu X., Wang X., Hu W. (2021). Outcomes of respiratory viral-bacterial co-infection in adult hospitalized patients. eClinicalMedicine.

[44] Bakaletz L.O. (2017). Viral-bacterial co-infections in the respiratory tract. Curr. Opin. Microbiol..

[45] Pacheco G.A., Gálvez N.M.S., Soto J.A., Andrade C.A., Kalergis A.M. (2021). Bacterial and Viral Coinfections with the Human Respiratory Syncytial Virus. Microorganisms.

[46] Bassis C.M., Tang A.L., Young V.B., Pynnonen M.A. (2014). The nasal cavity microbiota of healthy adults. Microbiome.

[47] Petat H., Schuers M., Le Bas F., Humbert X., Rabiaza A., Corbet S., Vabret A., Gouilh M.A., Marguet C. (2025). Characterizing acute respiratory infections in primary care for better management of viral infections. npj Prim. Care Respir. Med..

[48] Toro-Ascuy D., Cárdenas J.P., Zorondo-Rodríguez F., González D., Silva-Moreno E., Puebla C., Nunez-Parra A., Reyes-Cerpa S., Fuenzalida L.F. (2023). Microbiota Profile of the Nasal Cavity According to Lifestyles in Healthy Adults in Santiago, Chile. Microorganisms.

[49] Çelik M., Polat M.R., Avkan-Oğuz V. (2025). Diagnostic utility of rapid antigen testing as point-of-care test for influenza and other respiratory viruses in patients with acute respiratory illness. Diagn. Microbiol. Infect. Dis..

[50] Pérez-Ruiz M., Pedrosa-Corral I., Sanbonmatsu-Gámez S., Navarro-Marí M. (2012). Laboratory detection of respiratory viruses by automated techniques. Open Virol. J..

[51] Navarro-Marí J.M., Sanbonmatsu-Gámez S., Pérez-Ruiz M., De La Rosa-Fraile M. (1999). Rapid detection of respiratory viruses by shell vial assay using simultaneous cultura of HEp-2, LLC-MK2, and MDCK cells in a single vial. J. Clin. Microbiol..

[52] Weinberg A., Brewster L., Clark J., Simoes E., ARIVAC consortium (2004). Evaluation of R-Mix shell vials for the diagnosis of viral respiratory tract infections. J. Clin. Virol..

[53] Dunn J.J., Woolstenhulme R.D., Langer J., Carroll K.C. (2004). Sensitivity of respiratory virus culture when screening with R-mix fresh cells. J. Clin. Microbiol..

[54] Hou H., Wang T., Zhang B., Luo Y., Mao L., Wang F., Wu S., Sun Z. (2020). Detection of IgM and IgG antibodies in patients with coronavirus disease 2019. Clin. Transl. Immunol..

[55] ViVikerfors T., Lindegren G., Grandien M., van der Logt J. (1989). Diagnosis of influenza A virus infections by detection of specific immunoglobulins M., A, and G in serum. J. Clin. Microbiol..

[56] Phuong H.L., Nga T.T., Van Doornum G.J., Groen J., Binh T.Q., Giao P.T., Hung L.Q., Nams N.V., A Kager P., de Vries P.J. (2010). Viral respiratory tract infections among patients with acute undifferentiated fever in Vietnam. Southeast Asian J. Trop. Med. Public Health.

[57] Navarro-Marí J.M. (2016). Rapid diagnostic methods for acute viral respiratory infections. Enferm. Infecc. Microbiol Clin..

[58] Alonaizan F., AlHumaid J., AlJindan R., Bedi S., Dardas H., Abdulfattah D., Ashour H., AlShahrani M., Omar O. (2022). Sensitivity and Specificity of Rapid SARS-CoV-2 Antigen Detection Using Different Sampling Methods: A Clinical Unicentral Study. Int. J. Environ. Res. Public Health.

[59] CDC (2024). Overview of Influenza Testing Methods. https://www.cdc.gov/flu/hcp/testing-methods/index.html.

[60] Chartrand C., Tremblay N., Renaud C., Papenburg J. (2015). Diagnostic Accuracy of Rapid Antigen Detection Tests for Respiratory Syncytial Virus Infection: Systematic Review and Meta-analysis. J. Clin. Microbiol..

[61] Mahony J.B. (2008). Detection of respiratory viruses by molecular methods. Clin. Microbiol. Rev..

[62] Brittain-Long R., Westin J., Olofsson S., Lindh M., Andersson L.-M. (2010). Prospective evaluation of a novel multiplex real-time PCR assay for detection of fifteen respiratory pathogens—Duration of symptoms significantly affects detection rate. J. Clin. Virol..

[63] Martins Júnior R.B., Carney S., Goldemberg D., Bonine L., Spano L.C., Siqueira M., Checon R.E. (2014). Detection of respiratory viruses by real-time polymerase chain reaction in outpatients with acute respiratory infection. Mem. Do Inst. Oswaldo Cruz.

[64] Kumar A., Bahal A., Singh L., Ninawe S., Grover N., Suman N. (2023). Utility of multiplex real-time PCR for diagnosing paediatric acute respiratory tract infection in a tertiary care hospital. Med. J. Armed Forces India.

[65] Protocol: Real-Time RT-PCR Assays for the Detection of SARS-CoV-2. https://www.who.int/docs/default-source/coronaviruse/real-time-rt-pcr-assays-for-the-detection-of-sars-cov-2-institut-pasteur-paris.pdf.

[66] https://stacks.cdc.gov/view/cdc/88834.

[67] Pham V.H., Gargiulo Isacco C., Nguyen K.C.D., Le S.H., Tran D.K., Nguyen Q.V., Pham H.T., Aityan S., Pham S.T., Cantore S. (2020). Rapid and sensitive diagnostic procedure for multiple detection of pandemic Coronaviridae family members SARS-CoV-2, SARS-CoV, MERS-CoV and HCoV: A translational research and cooperation between the Phan Chau Trinh University in Vietnam and University of Bari “Aldo Moro” in Italy. Eur. Rev. Med. Pharmacol. Sci..

[68] Inchingolo A.D., Gargiulo C.I., Malcangi G., Ciocia A.M., Patano A., Azzollini D., Piras F., Barile G., Settanni V., Mancini A. (2022). Diagnosis of SARS-CoV-2 during the Pandemic by Multiplex RT-rPCR hCoV Test: Future Perspectives. Pathogens.

[69] Pham V.H., Pham H.T., Balzanelli M.G., Distratis P., Lazzaro R., Nguyen Q.V., Tran V.Q., Tran D.K., Phan L.D., Pham S.M. (2023). Multiplex RT Real-Time PCR Based on Target Failure to Detect and Identify Different Variants of SARS-CoV-2: A Feasible Method That Can Be Applied in Clinical Laboratories. Diagnostics.

[70] Van P.H., Thanh N.V., Ngoc T.V., Duy N.D., Huong L.T.T., Thuy C.T.M., Thao L.T.K., Thao N.T.H., Huong P.T., Camelia P.Q. (2018). Microbial Pathogens Causing Acute Lower Respiratory Infections in out Patients—The Preliminary Results from EACRI Study. Ho Chi Minh City Respiratory Society.

[71] Van Thao N.T., Tuan T.A., Van P.H., Vu L.T. (2025). Virus-induced asthma exacerbations in Vietnamese preschoolers. Ital. J. Med..

[72] Van P.H., An V.D.X., Quoc N.V., Binh P.T. The solution for the low-income countries to establish the automatic extraction of the nucleic acid from the clinical samples. Proceedings of the 14th Asian Congress on Medical Biotechnology and Molecular Biosciences.

[73] Van P.H., Thanh B.T., Quoc N.V., Viet N.Q., Huong P.T. Production and evaluation of the kit using magnetic silica coated nano-iron beads to extract the nucleic acid from different samples. Proceedings of the International Symposium on Antimicrobial Agents and Resistance (ISAAR) 2017.

[74] Thanh B.T., Van Sau N., Ju H., Bashir M.J.K., Jun H.K., Phan T.B., Ngo Q.M., Tran N.Q., Hai T.H., Van P.H. (2019). Immobilization of Protein A on Monodisperse Magnetic Nanoparticles for Biomedical Applications. J. Nanomater..

[75] Pham V.H., Nguyen T.V., Tran N.V., Nguyen D.D., Le H.T.T., Cao T.M.T., Le T.K.T., Nguyen T.H.T., Pham H.T., Quek C.Y.J. Microbial pathogens causing community acquired pneumonia in Vietnamese outpatients. Proceedings of the International Symposium on Antimicrobial Agents and Resistance.

[76] Pham H.T., Nguyen P.T.T., Tran S.T., Phung T.T.B. (2020). Clinical and Pathogenic Characteristics of Lower Respiratory Tract Infection Treated at the Vietnam National Children’s Hospital. Can. J. Infect. Dis. Med Microbiol..

[77] Lu L., Robertson G., Ashworth J., Hong A.P., Shi T., Ivens A., Thwaites G., Baker S., Woolhouse M. (2020). Epidemiology and Phylogenetic Analysis of Viral Respiratory Infections in Vietnam. Front. Microbiol..

[78] Obasi C.N., Barrett B., Brown R., Vrtis R., Barlow S., Muller D., Gern J. (2014). Detection of viral and bacterial pathogens in acute respiratory infections. J. Infect..

